# MDR-TB treatment as prevention: The projected population-level impact of expanded treatment for multidrug-resistant tuberculosis

**DOI:** 10.1371/journal.pone.0172748

**Published:** 2017-03-08

**Authors:** Emily A. Kendall, Andrew S. Azman, Frank G. Cobelens, David W. Dowdy

**Affiliations:** 1 Division of Infectious Diseases, Johns Hopkins University School of Medicine, Baltimore, Maryland, United States of America; 2 Department of Epidemiology, Johns Hopkins Bloomberg School of Public Health, Baltimore, Maryland, United States of America; 3 Amsterdam Institute for Global Health and Development, Academic Medical Centre, Amsterdam, Netherlands; Indian Institute of Science, INDIA

## Abstract

**Background:**

In 2013, approximately 480,000 people developed active multidrug-resistant tuberculosis (MDR-TB), while only 97,000 started MDR-TB treatment. We sought to estimate the impact of improving access to MDR-TB diagnosis and treatment, under multiple diagnostic algorithm and treatment regimen scenarios, on ten-year projections of MDR-TB incidence and mortality.

**Methods:**

We constructed a dynamic transmission model of an MDR-TB epidemic in an illustrative East/Southeast Asian setting. Using approximate Bayesian computation, we investigated a wide array of potential epidemic trajectories consistent with current notification data and known TB epidemiology.

**Results:**

Despite an overall projected decline in TB incidence, data-consistent simulations suggested that MDR-TB incidence is likely to rise between 2015 and 2025 under continued 2013 treatment practices, although with considerable uncertainty (median 17% increase, 95% Uncertainty Range [UR] -38% to +137%). But if, by 2017, all identified active TB patients with previously-treated TB could be tested for drug susceptibility, and 85% of those with MDR-TB could initiate MDR-appropriate treatment, then MDR-TB incidence in 2025 could be reduced by 26% (95% UR 4–52%) relative to projections under continued current practice. Also expanding this drug-susceptibility testing and appropriate MDR-TB treatment to treatment-naïve as well as previously-treated TB cases, by 2020, could reduce MDR-TB incidence in 2025 by 29% (95% UR 6–55%) compared to continued current practice. If this diagnosis and treatment of all MDR-TB in known active TB cases by 2020 could be implemented via a novel second-line regimen with similar effectiveness and tolerability as current first-line therapy, a 54% (95% UR 20–74%) reduction in MDR-TB incidence compared to current-practice projections could be achieved by 2025.

**Conclusions:**

Expansion of diagnosis and treatment of MDR-TB, even using current sub-optimal second-line regimens, is expected to significantly decrease MDR-TB incidence at the population level. Focusing MDR diagnostic efforts on previously-treated cases is an efficient first-step approach.

## Introduction

Multidrug resistant tuberculosis (MDR-TB) poses one of the greatest threats to TB control worldwide. Not only is the incidence of MDR-TB rising in many areas, curtailing our ability to meet ambitious targets for TB control, but the treatment of MDR-TB also threatens the economic solvency of national TB programs (NTPs), often accounting for more than 50% of annual NTP budgets [1]. Every year, the number of people developing incident active MDR-TB dramatically outpaces the number of people placed on MDR-TB treatment [2]. For example, in 2013, an estimated 480,000 people developed incident MDR-TB worldwide. Of these individuals, 300,000 were notified as having TB, but globally, fewer than 100,000 people were placed on treatment for MDR-TB [3], thereby leaving many people with infectious MDR-TB in the community.

This expanding pool of individuals with untreated MDR-TB represents an important and underappreciated source of transmission. “Treatment as prevention” has been emphasized for other infectious diseases (e.g. HIV [4] and malaria [5]) and, increasingly, for TB overall [6]. For MDR-TB, however, strategic discussions have largely focused on clinical outcomes [7] and cost [8]–with skepticism, given the low success rates of current MDR-TB drugs and treatment programs [9], about the extent to which expansion of current therapies can control MDR-TB [10]. To inform discussion of the potential preventive role of MDR-TB treatment, we constructed an epidemic model of MDR-TB in an illustrative high-burden East/Southeast Asian setting, calibrating to notification data from an illustrative country with high-quality data and epidemiologic analyses related to MDR-TB (Vietnam) and accounting for current low MDR-TB treatment coverage in the region. We used this model to evaluate the expected public health impact of increasing MDR-TB diagnosis and treatment, using either universal or risk-based drug susceptibility testing strategies, and compared this impact to the potential direct benefit of improving MDR-TB treatment outcomes among those currently receiving treatment.

## Methods

### Model structure

We used ordinary differential equations to develop a deterministic compartmental model of an adult pulmonary TB epidemic involving a drug-susceptible (DS, representing a weighted average of all non-MDR infections) and a drug-resistant (representing all MDR) *M*. *tuberculosis* strain. We modeled a setting in East/Southeast Asia, calibrating to age-adjusted WHO burden-of-disease estimates for Vietnam, an illustrative country with high-quality drug resistance survey data and related analyses but persistent low levels of MDR-TB treatment enrollment comparable to the global average [11], and with high TB and MDR-TB burdens not driven primarily by HIV or by unique high-intensity sources (e.g. prisons, mines).

**Fig 1:**
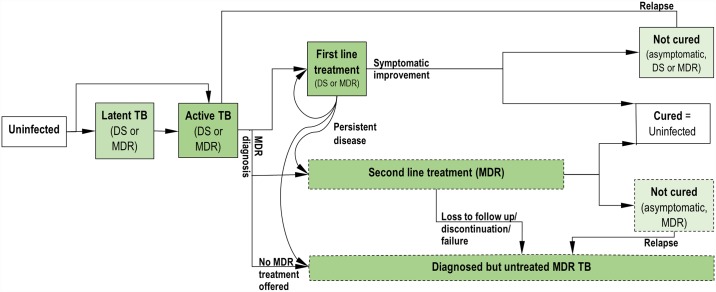
Model structure. Simplified diagram of modeled compartments. Separate compartments for never-treated and previously-treated individuals and for DS and MDR-TB at each stage are included in the model but not shown here. Also not shown: mortality (occurs at an increased rate during active disease) and spontaneous self-cure (can occur from any active disease or treatment compartment).

A complete description of the model system and calibration can be found in the SI. In brief, similar to prior published models [12,13], we model latent, early-active (subclinical), and active (care-seeking) disease states for each of DS and MDR strains (Fig 1). New infections result in either immediate early-active disease or latent disease with potential for later reactivation. Individuals with active TB are diagnosed with TB at a defined rate, and unless they are tested for and found to have MDR-TB, they immediately start first-line therapy. At baseline, only a fraction of individuals whose initial treatment fails or whose TB recurs receive drug susceptibility testing before retreatment, and only a fraction of those receiving MDR-TB diagnoses are offered MDR therapy; we calibrate MDR-TB under-diagnosis and under-treatment levels to Vietnam’s estimated MDR treatment initiations for 2013.

Potential outcomes of completed treatment include cure, suppression of disease with ongoing risk for relapse, and treatment failure with or without acquisition of resistance. Individuals also may be lost to follow-up, die, or spontaneously resolve their disease before completing treatment. Probabilities of each treatment outcome are based the TB strain (DS or MDR), the treatment regimen used (first-line or MDR), and the TB treatment status (treatment-naïve or previously-treated). We assume that MDR-TB patients treated with first-line therapy (except for those who die, are lost to follow-up, or spontaneously resolve during treatment) experience treatment failure and persist with active MDR-TB until death or spontaneous cure.

### Model calibration

We used an approximate Bayesian computation approach to capture uncertainty in the model parameters while remaining consistent with published WHO estimates for key epidemiological outputs (Table 1).

**Table 1:** Model calibration targets (based on WHO estimates for Vietnam).

Measure	Tolerance interval	Point estimate*	Reference
TB incidence, per 100,000 adults/year, 2000	185–338	256	[3,14]
TB incidence, per 100,000 adults/year, 2013	140–201	166	[3,14]
TB prevalence, per 100,000 adults/year, 2013	99–444	(242)	[3,14]
TB-attributable mortality, per 100,000 adults/year, 2013	16–34	(24)	[3,14]
Second-line treatment initiations, per 100,000 adults/year, 2013	0.7–1.6	(1.03)	[3]
DR fraction among newly-presenting TB cases, 2013	0.025–0.054	(0.040)	[3]
DR fraction among re-presenting TB cases, 2013	0.17–0.30	(0.23)	[3]

* Point estimates in parentheses were not explicitly used to fit our model. Incidence point estimates were used to fit the declining trend in total TB cases.

We added MDR-TB to a DS-only TB epidemic by allowing resistance to be acquired and transmitted beginning in 1960, and we modeled the region’s declining total TB incidence via a linear decrease in TB transmission efficiency since year 2000 (details in SI).

We estimated parameters by generating Latin hypercube samples [15] from a model parameter space of uniform or truncated-exponential prior distributions (that is, uniform distributions on either the arithmetic or logarithmic scale, depending whether uncertainty about the parameter was better represented on an absolute or a relative scale) representing literature-based uncertainty ranges (Table 2). We combined 1000 sets of general parameters with 1000 sets of drug-resistance-related parameters to generate 1,000,000 epidemic simulations for consideration, 8563 of which met all a-priori tolerance thresholds (Table 1) and were therefore selected for analysis.

**Table 2:** Model parameters.

Parameter	Mean estimate	Sampled Range	References
***General model parameters***			
Baseline mortality rate (15+ year olds) [year^-1^]	0.018	0.015–0.021	[14]
Additional mortality rate of untreated active TB [year^-1^]	0.2	0.1–0.4*	[16]
Transmission coefficient (secondary infections produced in a susceptible population, per active DS-TB case), in year 2000 [persons/year]	See methods		Fit to observed incidence trend (see S1 Text)
Probability of rapid progression after initial TB infection	0.11	0.04–0.18	[17]
Reduction in probability of rapid progression after second infection event, if already latently infected	0.43	0–0.86	[17,18]
Reactivation rate, latent to early active TB [year^-1^]	0.001	0.0005–0.002*	[18–21]
Progression rate from early-active (preclinical) to active TB [year^-1^]	1.4	0.7–2.8*	[22–24]
Infectiousness and mortality of early-active (preclinical) TB, relative to active TB	0.22	0.11–0.44*	[25]
Spontaneous TB cure rate [year^-1^]	0.18	0.13–0.23	[16,26]
TB diagnosis and treatment initiation rate, new patients [year^-1^] (x_N_)	0.6	0.3–1.2*	[27,28]
TB diagnosis and treatment initiation rate, previously-treated patients [year^-1^]	1.2	x_N_− 4x_N_	Model assumption
Bacteriologic response probability, first-line therapy, new patients (fraction of adherent patients who respond to treatment; includes those who will relapse)	0.98	0.96–1.0	[3]
Bacteriologic response probability, first-line therapy, retreatment patients (fraction of adherent patients who respond to treatment; includes those who will relapse)	0.94	0.88–1.0	[3]
Fraction of new patients retreated after failing first-line therapy, if determined not to have MDR-TB	1	-	Model assumption
Fraction of previously-treated patients retreated after failing first-line therapy, if determined not to have MDR-TB	0.5	0–1	Model assumption
Relapse risk after first-line therapy if no acquired resistance, new patients (ω_1N_)	0.04	0.02–0.08*	[29,30]
Relapse risk after first-line therapy if no acquired resistance, retreatment patients	†	ω1N – 0.12	[29,31]
Mean time to relapse, among patients who will relapse [years]	1.5	0.75–3.00*	[29]
Default risk during first-line therapy, new patients	0.02	0.01–0.04*	[3]
Default risk during first-line therapy, previously-treated patients	0.07	0.035–0.140*	[3]
Relapse risk after default from first-line therapy, new patients (*ω*_*δN*_)	0.2	0.1–0.4*	[30,32]
Relapse risk after default from first-line therapy, previously-treated patients	†	*ω*_*δN*_—0.8*	[30,32]
***Resistance-related parameters***			
Fitness of drug-resistant strain, relative to drug-susceptible strain fitness at year 2000	0.65	0.4–0.9	[33–35]
Risk of acquired resistance, DS-TB patients on first course of first-line therapy (α_N_)	0.006	0.003–0.012*	[30]
Risk of acquired resistance, DS-TB patients on retreatment with first-line therapy	†	α_N_− 0.060*	[36]
Drug susceptibility testing coverage, at end of failing retreatment	1	-	Model assumption
Drug susceptibility testing coverage, at end of failing initial treatment (s_fN_)	0.3	0–0.6	[3]
Drug susceptibility testing coverage, prior to retreatment (for treatment-experienced patients re-presenting to care)	†	0 –s_fN_	[3]
Drug susceptibility testing coverage, prior to initial treatment	0	-	Model assumption
Second-line treatment availability (fraction receiving treatment once MDR-TB is diagnosed)	0.6	0.2–1.0	[3]
MDR-TB relapse risk (fraction who relapse after completing suppressive second-line therapy)	0.07	0.02–0.12	[37–39]
Infectiousness during first six months of effective second-line treatment, relative to untreated active MDR-TB	0.05	0–0.10	[38,40]
Default risk, second-line therapy	0.16	0.08–0.32*	[41–43]
Bacteriologic response to MDR therapy (fraction of adherent patients who improve clinically and become culture-negative)	0.75	0.50–1.00	[38,41]

* parameter sampled from truncated exponential distribution (i.e., from uniform distributions on the logarithmic scale; all others are sampled from uniform distributions on an arithmetic scale)

^†^ parameter not independently estimated

We extended each data-consistent simulation forward in time to generate epidemic trajectories through year 2025. We modeled potential interventions as changes in model parameters starting in 2015.

### Sensitivity analysis

For MDR-TB incidence and mortality outcomes in 2025 under current practice and their relative reductions under the primary intervention of MDR screening and treatment among retreatment TB patients, we computed partial rank correlation coefficients [44] for each parameter after accounting for the effects of other parameters in the model, in addition to other sensitivity analyses mentioned above and described in full in the SI.

## Results

### Model fit to current TB epidemic

**Fig 2:**
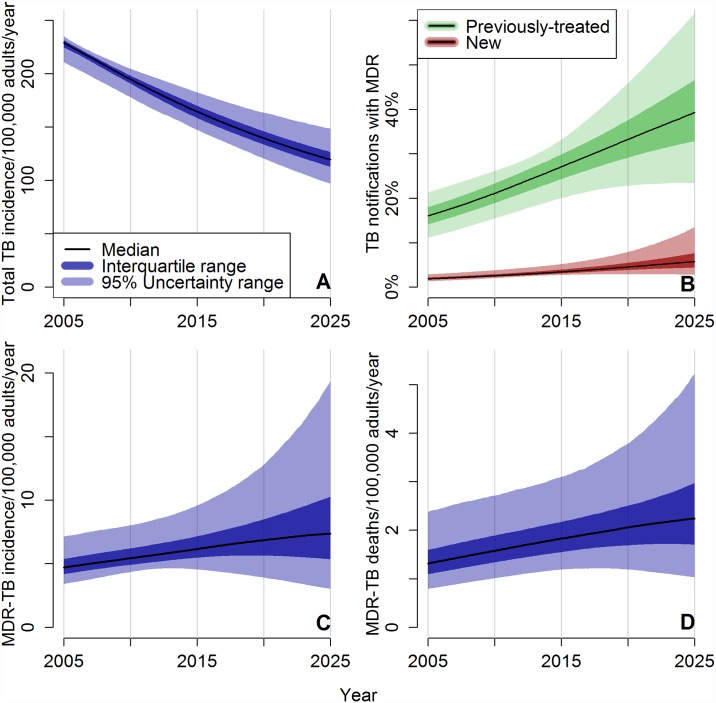
Model projections for East/Southeast Asian TB epidemic assuming continuation of current practice. Simulations are fitted to notification data for Vietnam through year 2013. Median and uncertainty ranges among the data-consistent projections are shown through year 2025, assuming unchanged diagnostic and treatment practices. The model assumes decline over time in the number of transmissions per infectious person-year, and therefore total TB incidence falls (panel A), but the fraction of both new and previously-treated patients who present to care with MDR-TB continues to rise (panel B), and MDR-TB incidence (panel C) and mortality (panel D) also rise until at least 2025 in the majority of data-consistent simulations.

The 8563 model trajectories that met tolerance thresholds reproduced TB incidence of 176 (95% UR 159–191) per 100,000 adults per year (Fig 2a), TB prevalence of 322 (UR 239–421) per 100,000 adults, and TB-attributable mortality of 27 (UR 18–34) deaths per 100,000 adults per year in 2013, reflecting the data constraints imposed.

While overall TB incidence declined between 2000 and 2013, MDR-TB incidence increased, from 3.8 (UR 2.6–6.1) cases/100,000 adults/year in 2000 to 5.9 (UR 4.6–8.8) in 2013. Among TB patients presenting to care, the fraction of notified patients who had MDR-TB (many of whose drug resistance remained undiagnosed) increased from 1.4% (UR 0.1–2.2%) of new patients and 12% (UR 8–17%) of previously-treated patients in 2000, to 3.0% (UR 2.5–4.5%) of new patients and 25% (UR 18–30%) of previously-treated patients in 2013. Mortality attributable to MDR-TB was 1.7 (UR 1.1–2.9) deaths per 100,000 adults per year in 2013 (Fig 2b–2d).

If current MDR-TB management continues (Fig 2), our model projects MDR-TB incidence rates of 7.4 (UR 3.0–19.4) per 100,000 in 2025, a 17% (UR -38 to +137%) increase in incidence compared to 2015, and mortality of 2.2 (UR 1.0–5.2) deaths per 100,000 due to MDR-TB in 2025, a 22% (UR -31 to + 122%) increase. By 2025, 5.7% (UR 2.7–13.5%) of new cases and 39% (UR 23–62%) of retreatment cases presenting to care would have MDR-TB. Despite declining total TB incidence, absolute MDR-TB incidence was projected to increase between 2015 and 2025 in 67% of model trajectories; it increased as a fraction of total incident TB in 94% of trajectories.

### MDR-TB treatment and transmission

We estimated that, for every incident MDR-TB case, 0.19 (95% UR 0.11–0.31) MDR treatment initiations occurred in 2013. The average duration of infectious TB until effective treatment initiation, death, or spontaneous resolution, was estimated to be 1.9 (UR 1.5–2.6)-fold longer for MDR-TB than DS-TB in 2013. During the time they were infectious, the average MDR-TB case transmitted 22 (UR 11–84) MDR-TB infections and ultimately produced 2.6 (UR 1.1–5.5) secondary cases of active MDR-TB disease, fueling a continuing epidemic through transmission alone. Of the transmissions occurring in 2013, 44% (UR 25–61%) arose from individuals with active MDR-TB who had presented for evaluation but had either not been diagnosed with, or not been offered appropriate treatment for, MDR-TB.

### Potential impacts of improved MDR-TB diagnosis and treatment

#### Expanding MDR diagnosis and treatment among retreatment patients

The primary intervention we considered was to (a) diagnose, via linear scale-up of drug-susceptibility testing between 2015 and 2017, all MDR-TB among all previously-treated patients re-initiating TB treatment, and to (b) initiate MDR therapy for 85% of those diagnosed with MDR-TB (that is, allowing for the same 15% initial loss to follow up typically observed among drug-susceptible TB patients [45]). We assumed continuation of typical present-day MDR-TB treatment outcomes for those treated.

**Fig 3:**
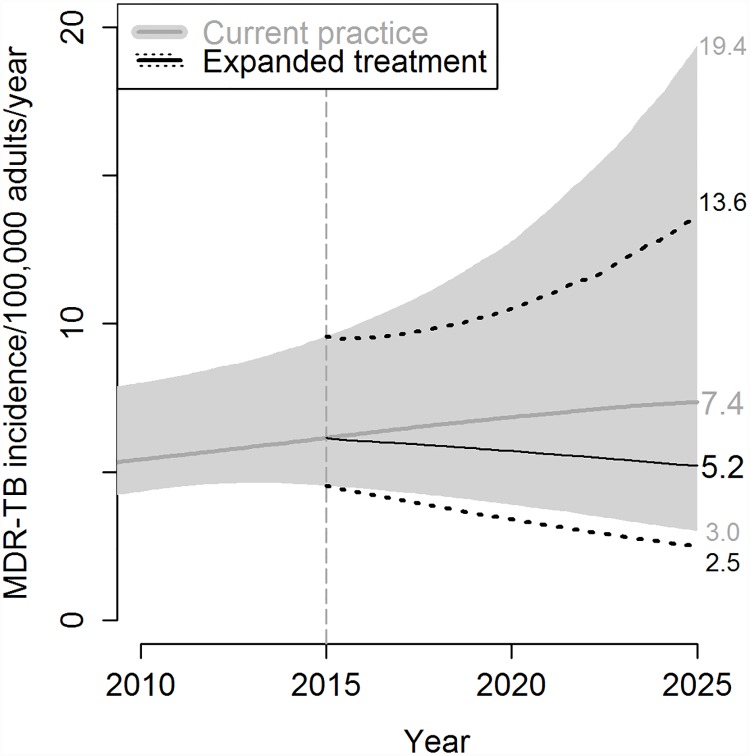
Impact of expanded drug-resistance diagnosis and second-line treatment availability. Under the intervention, use of drug susceptibility testing for previously-treated patients increases linearly from current levels in 2015 to 100% in 2017, and individuals found to have MDR-TB start second-line treatment, with allowance for 15% initial loss to follow up. Median and 95% uncertainty range values of MDR-TB incidence are shown, with continued current practice (gray) and under the intervention of expanded MDR-TB diagnosis and treatment (black with dotted 95% uncertainty range); their values in 2025 indicated numerically on the right. The outcome of this intervention in year 2025 is compared in Fig 4 with outcomes of other modeled interventions.

Of the retreatment patients receiving drug susceptibility testing in 2017 in this scenario, 27% (UR 19–35%) were expected to have MDR-TB at the start of the intervention. This expansion of drug susceptibility testing and accompanying MDR treatment among retreatment TB patients was estimated to reduce the average duration of an active MDR-TB episode by 12% (UR 3–25%), and correspondingly, to reduce the average numbers of secondary infections and secondary cases generated per MDR-TB episode by 13% (UR 3–26%) each. We project that within ten years, achieving this level of diagnosis and MDR treatment, together with MDR therapy for 85% of diagnosed cases, would reduce MDR-TB incidence in our East/Southeast Asian setting by 26% (UR 4–52%), and MDR-TB mortality by 32% (UR 9–59%), compared to no intervention (Fig 3).

#### Alternative intervention strategies

**Fig 4:**
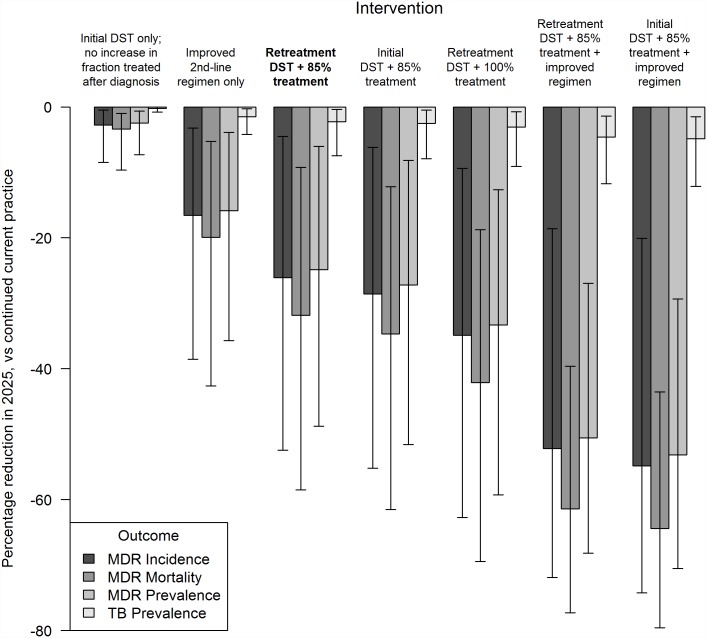
Impacts of primary and alternative interventions on multidrug-resistant tuberculosis (MDR-TB) epidemic in 2025. Drug susceptibility testing is performed either at current levels, or in all retreatment patients implemented (the primary intervention, shown in bold; linear scale-up completed by 2017), or in all patients prior to initial treatment (linear scale-up completed by 2020). Enrollment on appropriate MDR-TB treatment occurs either at current levels, or in 85% of diagnosed patients (allowing typical initial loss to follow up), or in 100% of diagnosed patients. For the “improved second-line regimen”, for MDR-TB patients’ adherence, cure, relapse, and time to non-infectiousness are equivalent to the standard first-line regimen outcomes for drug-susceptible TB patients. Error bars represent 95% uncertainty ranges among model simulations.

Up-front drug susceptibility testing in all patients prior to initial TB treatment (implemented as linear scale-up for retreatment patients over a two-year period and for new patients over a five-year period) leads to earlier diagnosis of MDR-TB for patients who, under the primary intervention discussed above, would have to fail first-line treatment before their MDR-TB was diagnosed. However, this up-front drug susceptibility testing (when combined with the same improved (85%) level of MDR treatment initiation after diagnosis as above) averts relatively little additional MDR-TB transmission and offers only a slight advantage over the strategy focused on retreatment patients (a 29% (UR 6–55%) rather than 26% incidence reduction) (Fig 4). Continuing to target only previously-treated patients as in the primary intervention, but focusing on linkage to care and thus completely eliminating the assumed 15% initial loss to follow-up, could provide a somewhat larger benefit, reducing MDR-TB incidence by 35% (UR 9–65%) compared to baseline projections (Fig 4).

An innovation that could increase both the feasibility and the impact of MDR-TB treatment scale-up is a novel MDR treatment regimen with shorter duration, all-oral administration route, and enhanced potency. An MDR regimen with cure rate, relapse rate, rapid elimination of infectiousness, and loss to follow up all equivalent to current first-line therapy could reduce MDR-TB incidence in 2025 by 17% (UR 3%-39%) compared to continued current practice even at current low levels of MDR-TB diagnosis and treatment coverage. If the primary intervention of 100% drug susceptibility testing and 85% treatment initiation for retreatment patients with MDR-TB were implemented using such a regimen, then our model projects a 52% (UR 19–72%) reduction in year-2025 MDR-TB incidence and a 61% (UR 40–77%) reduction in year-2025 MDR-TB mortality, relative to projections under continued current practice; also providing drug susceptibility testing and appropriate treatment to new TB patients increases this impact of an improved drug regimen to a 55% (UR 20–74%) MDR-TB incidence and 64% (UR 44–80%) MDR-TB mortality reduction (Fig 4).

### Sensitivity analysis

**Fig 5:**
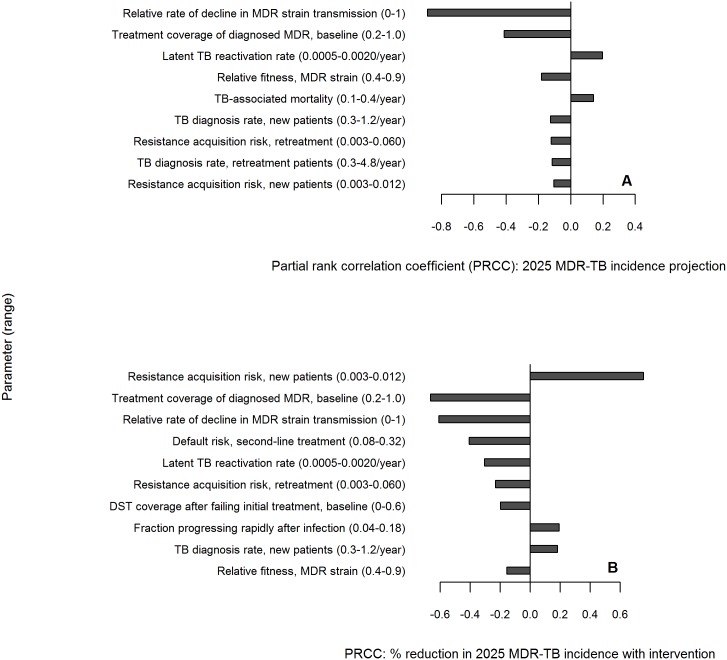
Multivariable sensitivity analysis. Partial rank correlation coefficients > 0 indicate that as the parameter of interest increases (after correction for the other parameters), the projected absolute MDR-TB incidence in 2025 under current practice increases (panel A), or the magnitude of the reduction in MDR-TB incidence under the primary intervention (85% MDR treatment coverage among retreatment patients) increases (panel B). Negative values likewise reflect negative correlation between parameter and output. “Relative rate of decline in MDR strain transmission” refers to the decrease in MDR-TB transmissions per infectious person-time, as DS-TB transmissions decrease to model declining TB incidence (methods are described further in S1 Text).

Both baseline projections and intervention impacts were heavily dependent on the transmission efficiency of the MDR strain relative to the DS strain, the fraction of known MDR-TB being treated currently, and the latent TB reactivation rate (as stopping transmission eliminates only recently-transmitted TB infections). The intervention’s impacts were also sensitive to the propensity for DS-TB patients to acquire resistance during treatment, and to characteristics of MDR therapy including the associated probabilities of cure, loss to follow up, and relapse (Fig 5). Results of other sensitivity analyses are included in S1 Text and S1 Table.

## Discussion

By simulating present-day MDR-TB care, we highlight an opportunity to redirect the MDR-TB epidemic within ten years by diagnosing and treating MDR-TB within a readily-identifiable set of high-risk patients. We demonstrate that, by meeting the Global Plan’s 2015 target of 100% drug susceptibility testing before retreatment [46] and by providing MDR therapy to diagnosed individuals (even assuming existing pretreatment losses to follow up and low treatment success rates), an upward trend in MDR-TB could be reversed, and incidence in 2025 could be reduced by approximately one quarter relative to current practice. Furthermore, these projected gains could be doubled if MDR-TB could be treated with a regimen as effective and tolerable as the current first-line regimen.

This profound projected impact of MDR-TB treatment as prevention reflects the large number of MDR-TB cases who currently remain undiagnosed and/or untreated for MDR-TB, and therefore presents a huge challenge as well as opportunity. The numbers of new MDR-TB cases and of prescribed treatments in Vietnam in 2013 were consistent with more than five times as many people developing MDR-TB as initiating appropriate MDR therapy [11], and due to limits in both diagnostic test availability and treatment capacity, similar low treatment coverage is widespread among high-burden countries [47]. Some who go without treatment die or spontaneously improve, but the remainder comprise a growing reservoir of transmission; our model suggests that the average MDR-TB case ultimately generates two or more secondary cases of active MDR-TB. Despite our model’s assumptions that drug-resistance mutations reduce transmissibility [35] and that transmission of both strains is declining over time (reflecting, for example, improving economic conditions), the current level of transmission is sufficient to sustain and even grow an MDR-TB epidemic for the next decade and beyond.

Previous mechanistic models have noted the importance of transmission in sustaining MDR-TB epidemics [48,49]. Other models have evaluated the impact of expanded drug susceptibility testing (e.g. GeneXpert use) and associated MDR-TB treatment. In the context of HIV-driven TB epidemics, such models have reached varying conclusions with regard to whether expanded MDR-TB diagnosis and treatment can produce sustained reductions in MDR-TB cases [13,50]. For MDR-TB epidemics not driven by HIV, a recent analysis in India has shown the potential for increased MDR-TB detection to prevent up to 25% of cumulative MDR-TB cases over 10 years, even in absence of concomitant improvements in average MDR treatment outcomes [51]. Our model, by explicitly focusing on the pool of untreated MDR-TB cases who contribute to ongoing transmission, reaches a similar conclusion: leveraging new diagnostic tools to expand treatment coverage even without waiting for better regimens, can prevent a quarter of new MDR-TB cases a decade from now, while a better regimen, if both short and effective, could double that impact to prevent more than half of cases.

Our model suggests that the majority of impact can be achieved by diagnosing and treating drug resistance only in those TB patients who have been treated for TB in the past (about 10% of all TB cases). This highlights the fact that most treatment-naïve individuals with primary, transmitted MDR-TB ultimately present as “previously treated” TB after first-line therapy fails, and that the duration of inappropriate first-line treatment is short relative to the total time that they remain infectious. Because of this double-counting of transmitted MDR-TB cases as both new TB notifications (with undiagnosed MDR) and then as retreatment TB notifications, the ratio of transmitted to treatment-acquired MDR-TB may be higher than a simple comparison of MDR prevalence in new versus previously treated TB cases would suggest. A high transmitted proportion among MDR-TB cases allows treatment, by averting transmission, to have greater population-level impact than might otherwise be expected: because of the secondary cases prevented, even current regimens that only cure roughly half of the patients they treat may nevertheless cure or prevent an average of more than one MDR-TB case per treatment course.

Although the logistical and infrastructure challenges of MDR detection and treatment scale-up remain substantial, the gains we model do not require drug susceptibility testing for all patients, nor do they depend on successfully treating individuals with “chronic” MDR-TB who often have failed multiple treatments and may respond poorly to therapy due to advanced disease or additional acquired resistance. Waiting until patients with primary MDR-TB fail therapy or present to care for the second time delays MDR treatment initiation, and also prioritizing up-front drug susceptibility testing for high-risk new patients such as MDR-TB contacts is advisable [11], but a strategic focus on first diagnosing and treatment MDR in previously-treated and other high-risk patients first may help resource-constrained programs to scale up MDR-TB treatment more rapidly over the next few years. It is important to note, however, that we did not model possible effects of an initial inappropriate course of first-line treatment on amplifying resistance; in particular, if failed treatment of MDR-TB with the first line regimen selects resistance to pyrazinamide also, and if this worsens subsequent second-line treatment outcomes, then identifying and appropriately treating MDR-TB patients when they first present to TB care could have greater advantages than our model estimates [52]. Under either an initial or a retreatment-only screening strategy, an MDR treatment regimen with better tolerability, efficacy, and ease of administration could add substantial value by improving the low cure rates of current MDR regimens, potentially doubling the impact of the same expansion in MDR detection and treatment coverage, and could also make this MDR treatment scale-up easier.

Although our model highlights that future MDR-TB epidemic trajectories cannot be predicted with certainty, our sensitivity analyses identify data elements that could refine those predictions. Better understanding of reactivation rates and of the proportion of MDR-TB cases resulting from recent transmission would clarify the time scale over which interrupting transmission can be expected to reduce incidence. More precise delineation of current MDR outcomes, of the relative transmissibility of drug-resistant TB strains, and of the stages of diagnosis/treatment at which MDR-TB cases are currently being missed, would improve our ability to predict what types of interventions will have greatest impact.

Like all models, ours has limitations arising from simplifying assumptions. In this initial projection of the population impact of widespread MDR-TB treatment, we assume homogeneity of host susceptibility (e.g. we do not differentiate East/Southeast Asia’s relatively small HIV-infected population), treatment responses, geographic MDR-TB case distribution, and (apart from the DS/MDR dichotomy) strain transmissibility and virulence. Future models could consider the potential augmented impact of interventions targeted to high-risk groups. We also assume perfect sensitivity of drug susceptibility testing and do not model specific diagnostic algorithms, leaving open the question of how to implement the modeled increases in drug susceptibility testing. Finally, although we conservatively assume that patients who have failed or stopped MDR therapy become incurable, we do not explicitly model second-line drug resistance and how treating more MDR-TB patients might increase the prevalence of such extended resistance [53].

Under current practices, in which the majority of individuals with MDR-TB remain untreated, the proportion (and likely the absolute number) of MDR-TB cases in many countries is expected to increase over the next decade and beyond. Our model suggests, however, that consistently identifying and treating MDR-TB among previously-treated TB patients, starting immediately, could reduce the incidence of MDR-TB in 2025 by more than 25% compared to expectations under current practice—or by more than 50% if paired with a highly-effective new MDR treatment regimen. Expanding MDR-TB detection and treatment will require investments in infrastructure and safeguards against second-line drug resistance, but it is key to effectively preventing MDR-TB transmission and reversing the tide of existing MDR-TB epidemics.

## Supporting information

S1 Text Supplementary methods and results. Includes additional description of model and of modeled interventions, as well as additional model calibration and sensitivity analysis results.
(DOCX)

S2 Text Model equations. Annotated differential equations specifying transmission model.
(PDF)

S1 Table Sensitivity analysis—Comparison with alternative model assumptions. (DOCX)

S1 Fig Parameter distributions. Prior distributions (among all parameters sets generated for consideration by Latin hypercube sampling) and posterior distributions (among epidemic trajectories that were accepted as consistent with present-data notification data) of key model parameters. Most parameters’ sampled ranges were represented evenly among the accepted simulations (blue curves, most of which are similar to the red), but a few parameters (notably the extent of second-line treatment coverage and the relative fitness of the MDR strain) exerted greater influence on the fit of simulations to notification data.
(JPG)
