# Supplementary material for: MDR-TB treatment as prevention: The projected population-level impact of expanded treatment for multidrug-resistant tuberculosis
Source: PLoS One. 2017 Mar 8;12(3):e0172748. doi: 10.1371/journal.pone.0172748 (PMC5342197; doi:10.1371/journal.pone.0172748)
Supplement: S1 Text — Includes additional description of model and of modeled interventions, as well as additional model calibration and sensitivity analysis results. (DOCX) [file pone.0172748.s001.docx]

**S1 Text: Supplementary methods and results**

MDR-TB Treatment as Prevention: The Projected Population-Level Impact of Expanded Treatment for Multidrug-Resistant Tuberculosis

Emily A. Kendall, Andrew S. Azman, Frank G. Cobelens, David W. Dowdy

**Full narrative description of model:**

We constructed a deterministic compartmental model of a TB epidemic with two strains of *M. tuberculosis*: a drug-susceptible (DS) strain, and a drug-resistant (MDR) strain resistant to at least rifampin.

Uninfected individuals face a continual risk of infection from each strain proportional to the prevalence in the population of active TB of that strain, weighted by infectiousness of the stage of active disease for each infectious individual, and reduced for the MDR strain compared to the DS strain because of an assumed loss of transmission fitness associated with drug resistance.

To conservatively estimate the potential impact of second-line treatment, we consider active, fully-infectious TB to develop after an early (subclinical) active phase with reduced infectiousness (assumed to be equivalent to smear-negative pulmonary TB) during which individuals to not seek medical treatment (1). Successful infection events can result either in immediate “rapid progression” to this subclinical phase (and ultimately to symptomatic disease), or in latent infection, which in turn has an annual risk *r* of progression to early active disease at any point in a person’s lifetime (“reactivation”). From early active disease, infections progress to active disease at rate *a*.

Individuals with latent infection may be super-infected with the same strain or with the other strain. When superinfection occurs, the probability of rapid progression to early active disease is reduced by a fraction λ compared to the probability of rapid progression in a first infection. If the super-infecting strain is different from the original latent strain and does not result in rapid progression, then rather than resulting in two coexisting infections, the two strains compete, with odds of replacement proportional to the fitness of the new strain relative to the old.

In addition to the baseline mortality rate for adults age >15, individuals with active TB face an additional mortality rate *μ_tb_*, which is reduced in proportion to infectiousness for disease states with reduced infectiousness. Individuals with any active TB also have the possibility of spontaneous cure, which occurs at a specified rate *η*.

Once individuals develop [fully] active disease, they begin to be diagnosed and (unless known or found to have MDR-TB) started on first-line treatment at a specified rate (*x_N_* for new patients, *x_P_* for previously-treated patients). In our baseline model, we assume there is no up-front drug susceptibility testing, so all new patients are started initially on first-line treatment, but we also model interventions involving drug susceptibility testing prior to initial treatment.

First-line treatment, appropriate for DS-TB, lasts six months and, may be effective (probability *σ_N_* or *σ_P_***)** or ineffective at suppressing DS-TB and leading to culture conversion. Because the time to noninfectiousness on effective first-line therapy is negligible compared to other relevant time periods, patients on effective first-line treatment are modeled as becoming immediately noninfectious (2). Individuals may complete treatment or be lost to follow up after starting treatment (probability δ_1N_ or δ_1P_ per treatment course, or δ_2_ for MDR treatment), and they also continue to have a possibility of death or spontaneous resolution throughout the treatment course.

At the end of effectively suppressive treatment, individuals may be cured (but susceptible to reinfection), or with probability *ω_N_ or ω_P_*  they may become noninfectious and asymptomatic but remain at risk for relapse, which then occurs at a specified rate *τ_ω_*. Finally, during treatment of DS-TB, a fraction (*α_N_* or *α_P_)* of patients acquire resistance, resulting in immediate active MDR-TB in those failing treatment, and in relapse soon after treatment completion in those who would otherwise be cured. Probabilities of first-line treatment failure, relapse, resistance acquisition, and loss to follow up are higher for previously-treated patients than for new patients.

For patients on suppressive first-line therapy who are lost to follow up, the probability of relapse (*ω_δN_* or *ω_δP_*) is based on the fraction of the treatment course that the average person lost to follow up completes (3) and the average relapse rates for rifampin-based regimens of that duration (4). Because most acquisition of resistance likely occurs early in treatment when the bacterial burden is high (5), probabilities of acquired resistance in individuals who are lost to follow up are assumed to be equal to those of individuals who complete the treatment course.

Individuals whose first-line treatment is ineffective at suppressing their disease (which includes some individuals with DS-TB and all individuals with MDR-TB) and who do not spontaneously cure will experience treatment failure. They remain infectious and symptomatic; our primary model assumes they remain as infectious as without treatment, but in sensitivity analysis we consider the possibility that their infectiousness is partially (75%) reduced while on ineffective treatment. We assume that individuals who fail first-line therapy but remain linked to care remain at least partially symptomatic and thus receive a second treatment course and/or drug susceptibility after initial failure. After failing a retreatment course, a fraction *γ_P_* either receive drug susceptibility testing or are immediately restarted on first-line therapy, while the remainder revert to active DS-TB.

Our model accounts for the present low number of second-line treatment initiations per incident MDR-TB case through a combination of suboptimal diagnosis of drug resistance and suboptimal treatment of identified MDR-TB cases. Fractions *s_fN_* or *s_pP_* of TB patients, respectively, receive drug susceptibility testing upon failing initial treatment or prior to retreatment after relapse/reinfection; we assume that no treatment-naïve individuals receive drug susceptibility testing before their initial course of treatment *(s_pN_* = 0) and that all MDR is diagnosed once a patient fails a second course of first-line therapy (*S_fP_* = 1). Once MDR is diagnosed, only a fraction *b* of individuals diagnosed with MDR-TB are offered second-line therapy.

MDR therapy, when effective, reduces the infectiousness of MDR-TB substantially for the first six months and then renders patients non-infectious for the remainder of a 24-month treatment course (6). After two years, most individuals who had responded to MDR therapy are cured, but a fraction *ω_2_* will relapse to active MDR-TB at rate *τ_ω_*. Those MDR-TB patients who fail to respond to second-line therapy remain fully infectious and symptomatic. Loss to follow up during MDR therapy (in a fraction *δ*_2_ of patients) and discontinuation of ineffective MDR therapy are both assumed to occur at six months. Individuals who are lost to follow up, fail, or relapse after MDR therapy, along with those who are diagnosed with MDR-TB but not offered second-line therapy, remain infectious until they die or are spontaneously cured. We consider appropriate treatment for MDR-TB (“MDR therapy”) to reflect the existing mix of second-line regimens in an illustrative East/Southeast Asian setting, including any subsequent adjustments made based on toxicity, drug susceptibility results, etc. We assume therefore that each patient has one opportunity to undergo MDR therapy – an opportunity that includes all adjustments to the initial regimen.

Besides rifampin resistance or MDR, other resistance patterns such as isoniazid monoresistance and second-line drug resistance are not explicitly modeled, but are reflected in higher probabilities of first-line treatment failure, relapse, and resistance acquisition for previously-treated patients than for new patients, and in lack of treatment for patients who have failed second-line therapy.

We model a population of adults aged >15 years, with new (15-year-old) individuals entering at each time step to replace deaths and maintain constant population size. These 15-year-olds enter the population with latent DS and MDR-TB prevalence calculated based on their cumulative exposure to each strain during childhood. This prevalence is estimated based on the current active TB incidence and the mean transmission coefficient over the prior 15 years, *β_S_*(*t*-(15/2)) or *β_R_*(*t*-(15/2)). No individuals enter with active or previously-treated TB.

The model is specified in differential equations at the end of this supporting information. The model system was implemented in R version 3.1.1 (14), using the deSolve package (15) with a time step of 0.1 year.

**Additional model calibration details**

Incident TB in our model was defined as any transition from a noninfectious to an infectious TB disease state. MDR-TB incidence also included transitions from active DS-TB to active MDR-TB when resistance was acquired during treatment. TB prevalence included all active TB, early-active TB, and TB currently receiving effective or ineffective treatment. TB-related deaths in HIV-infected individuals were included in TB mortality. Retreatment cases included all relapses, reinfections in previously-treated individuals, and retreatments after failure or loss to follow up. Notifications were distinct from incidence, and occurred each time an individual either presented to care and was diagnosed with TB or restarted treatment after an ineffective treatment course. Some incident cases thus generate multiple notifications and treatments, first as new patients and later as previously-treated patients.

We derived incidence, prevalence, and mortality estimates for our adult-only population by multiplying WHO’s population-wide estimates by 0.89, the estimated fraction of total TB burden occurring in adults based on the relationship between TB incidence and pediatric TB fraction described by Jenkins et al. (7), and then dividing by the fraction of the population that was >15 years old in the year under consideration (8). We assumed that the same fraction of total TB was drug-resistant in adults as in children, and that all notified MDR cases were adult.

In our first calibration step, involving a DS-only model, we determined the relationship at equilibrium between the transmission coefficient *β_0_* and the simulated TB incidence for each of 1000 LHS-generated sets of parameters. Based on that relationship, we then determined the rate of linear decrease *d* in the transmission coefficient *β_S_(t)* that would produce the total TB incidence observed in Vietnam in years 2000 and 2013. We then ran each model forward in time, starting at equilibrium in year 2000, with a declining *β_S_(t)* from 2000 to 2013. Parameter sets that led to simulated TB prevalence and mortality rates in 2013 within the WHO-data-based (9) uncertainty ranges (Table 1) were then selected for calibration of a model with MDR-TB. In a sensitivity analysis, we assumed the decline was due to lower reactivation, rather than transmission, rates (details below).

We chose our parameter samples in such a way that excluded impossible or highly unlikely combinations (e.g., probabilities greater than one, or better treatment success for previously-treated than for new patients).

In the second step of model calibration, we performed LHS to generate 1000 sets of drug-resistance-related parameters to add to each accepted DS-TB-only simulation. For each set of resistance-related model parameters, we took a DS-only TB epidemic at equilibrium with transmission coefficient *β_0_*, then allowed acquisition and transmission of resistance to begin and ran the model forward for 40 years with constant transmission coefficient *β_0_*, to generate an MDR-TB-containing human population for year 2000. The model then continued forward from year 2000, with *β_S_(t)* declining at rate *β_0_d* as above, and with the MDR strain transmission coefficient declining from *f_R_* *β_0_* at the slower rate, *f_R_β_0_ed*, where *e* was sampled over the range 0 to 1. Trajectories that produced values in year 2013 for TB incidence, TB prevalence, TB-attributable mortality, MDR treatment initiations, MDR prevalence among new patients presenting to care, and MDR prevalence among previously-treated patients at time of retreatment, which were all within uncertainty ranges for 2013 in Vietnam based on WHO data, were then selected for analysis. Because the WHO publishes only a point estimate of MDR treatment initiations, we constructed an uncertainty range around the point estimate of comparable width to those for mortality and for MDR prevalence.

Unless otherwise noted, outcomes are reported as a median result from the 8563 accepted simulations, with 95% uncertainty ranges (UR) in parentheses representing the 2.5^th^ and 97.5^th^ percentile results among accepted simulations.

To confirm that our Latin hypercube samples of the parameter space were sufficiently large, we doubled the number of sampled parameter sets to 2,000,000 by doubling the initial LHS size and verified that for key results, median estimates did not change by a factor of more than 2% and endpoints of 95% uncertainty ranges by a factor of more than 5%. The estimated median number of secondary infections produced by each MDR case did decrease by 6% with this doubling, and the upper bounds of the 95% confidence intervals for numbers of secondary total and active infections decreased by 14% and 18% respectively, but the variations in all other results reported in this manuscript were within these target ranges.

We estimated average duration of active TB in 2013 as prevalence/(incidence – d (prevalence)/dt) for each strain. We estimated the number of secondary infections produced by multiplying this duration, weighted by infectiousness of each stage of disease, times the force of infection of that strain, times the susceptible population size plus the latent-protection-adjusted latently-infected population size. We then estimated the number of those that became active cases by multiplying the total number of infections by the sum of the fraction rapidly progressing, and the life expectancy times the reactivation rate.

**Intervention details**

The primary intervention we modeled, “retreatment MDR screening and treatment,” was diagnosis of all MDR-TB (i.e., performing drug susceptibility testing, assuming perfect accuracy) in previously-treated patients only, implemented as a linear increase in drug susceptibility testing from baseline levels in 2015 to full coverage of previously-treated patients in 2017, and combined with MDR initiation for 85% of those diagnosed with MDR-TB (allowing for 15% pre-treatment loss to follow-up as typically observed in DS-TB (10).) We assumed that MDR treatment outcomes remained unchanged from baseline with this intervention: i.e., a median 16% of patients discontinuing treatment due to toxicity or loss to follow-up, 25% treatment failure among those who remained on treatment (with a sensitivity analysis in which treatment failure is reduced by half), and 7% relapse among those who appeared to have been treated successfully.

In secondary analysis, we also considered alternative interventions involving combinations of the following changes: drug susceptibility testing before initial treatment (offered to all patients by 2020, and implemented as a linear increase in drug susceptibility testing coverage for new patients from current levels to 100% over five years between 2015 and 2020, and as a linear increase for retreatment patients from current levels to 100% over two years from 2015 to 2017); improved pre-treatment loss to follow up (resulting in treatment initiation for 100% rather than 85% of those diagnosed with MDR-TB); and improved MDR regimens (resulting in improvement in the probabilities of loss to follow up, failure, and relapse associated with MDR therapy to mirror the current first-line treatment outcomes of new DS-TB patients).

**Additional sensitivity analyses**

To evaluate the impact of our assumption that patients remained fully infectious while on ineffective treatment, we reduced the infectiousness (compared to that of untreated active TB) of both DS and MDR patients on ineffective first- or second line treatment from 100% to 25%. We sampled 10,000 parameter sets with this reduced infectiousness but with other parameters (including the mortality associated with active TB during ineffective treatment) unchanged. We compared year-2025 MDR-TB incidence projections, with existing control measures and under each of the two DST and second-line treatment interventions (in retreatment patients, and in all patients), to the projections of the primary model.

We also considered an alternate model in which the observed downward trend in total TB incidence arises from a decline in the reactivation rate of latent TB instead of a decline in the transmission rate. We tested 200 sets of general parameters, each paired with 200 sets of MDR-TB-related parameters using this model. We again compared the two models’ year-2025 MDR-TB incidence projections, under existing control measures and under each of two interventions.

To evaluate sensitivity of our results to the assumption that the probability that MDR-TB treatment will be curative is the same whenever it is started (e.g. regardless of whether a patient had first been treated with inappropriate first-line therapy), we simulated the intervention of up-front DST (linear scale-up to 100% by 2025) with a 50% reduction in the second-line treatment failure probability for those MDR-TB patients who had never previously been treated for TB. We compared the resulting impacts on absolute and relative DR TB incidence and mortality in 2025 with the scenario in which second-line failure rates are unchanged in new vs retreatment MDR patients.

Finally, because the model, when calibrated to Vietnam TB incidence estimates, yielded TB prevalences in the higher part of the uncertainty range for the observed TB prevalence in Vietnam (2007 (11)), we considered the possibility that our model overestimated the average duration of active TB. We recalculated our results using only those accepted parameter sets that produced an average DS TB duration of less than the 50th percentile prediction among all accepted parameter sets.

**Supplemental Results - Model calibration**

In the first stage of model calibration, the simulations resulting from 421 of 1000 general TB parameter sets met selection constraints. After each set of general TB parameters was paired with 1000 sets of MDR-TB-related parameters, 8563 of the resulting simulations (arising from 368 of the DS-only simulations) met all selection criteria for year 2013, including those for MDR-TB prevalence and second-line treatment initiations, and were therefore used for analysis of future epidemic trajectories and intervention impacts. Values of parameters such as MDR strain fitness, TB mortality, and baseline second-line treatment availability were critical to whether simulations met selection constraints, while for other parameters, accepted trajectories represented the full range of the sampled prior distribution (S1 Fig).

Because we allowed for variation in the relative importance of missed diagnoses and diagnosed-but-untreated cases in producing this low number of MDR-TB treatments, DST use ranged from 0 to 60% (median 32%) at the time of initial treatment failure and from 0 to 59% (median 13%) upon presentation with recurrent TB, while second-line therapy was given to 20 to 100% (median 48%) of individuals who had been diagnosed with drug resistance. Among prevalent MDR-TB cases in 2013, 7.4% (UR 3.7-13.6%) were currently on second line therapy, 6.0% (UR 1.9-12.4%) had previously received second line therapy but failed, were lost to follow up, or relapsed, and 86% (UR 77-93%) had never received second-line therapy.

**Supplemental Results - Sensitivity analysis**

In partial rank correlation analysis, as noted in the main text, both baseline projections and intervention impacts were heavily dependent on the transmission efficiency of the MDR strain relative to the DS strain, the fraction of known MDR-TB being treated currently, and the latent TB reactivation rate (as stopping transmission eliminates only recently-transmitted TB infections), and the intervention’s impacts were also sensitive to the propensity for DS-TB patients to acquire resistance during treatment, and to characteristics of MDR therapy including the associated probabilities of cure, loss to follow up, and relapse (Figure 5). When the rate of reactivation from latent to active disease, rather than the transmission coefficient, was reduced linearly over time to replicate E/SEA’s observed downward TB incidence trend, baseline projections of MDR-TB incidence were similar to those obtained with the declining transmission coefficient. However, because the contribution of reactivated remote infections diminished over time in the declining-reactivation-rate model, ongoing transmission accounted for a larger proportion of incident TB, and therefore interventions to diagnose and treat individuals with infectious MDR-TB had an even larger impact than in the declining-transmission-coefficient model (S1 Table). The impacts of interventions differed significantly between the models (p<0.00001 for two-sample t test), and our primary model generated the more conservative estimates of potential impacts of increased MDR-TB diagnosis and treatment.

Reducing the relative infectiousness of individuals on ineffective treatment from 100% to 25%, compared to the infectiousness of individuals on no treatment, narrowed the uncertainty ranges but had little impact on median projections of MDR-TB incidence under current care or improved diagnosis and treatment (S1 Table).

The parameter sets corresponding to the lower 50% of DS-TB duration had lower TB transmission efficiency, higher TB-associated mortality, and faster progression both from subclinical to fully active disease and from active disease to diagnosis. Considering only these simulation reduced our estimate of total pulmonary TB prevalence to 289 (UR 233-329) per 100,000 adults, with little change in other estimates including TB incidence (175 (UR 159-190) per 100,000 per year), TB mortality (26 (UR 17-34) per 100,000 per year), MDR-TB incidence (5.7 (UR 4.6-8.6) per 100,000 per year), or the percent with MDR-TB among new TB notifications (3.0% (UR 2.5-4.5%)) or retreatment TB notifications (24% (UR 18-30%)) in 2013, nor in projected MDR-TB incidence (7.1 (UR 3.0-18.1)) or MDR-TB percent among new (5.6% (UR 4.3-7.6%) or retreatment (39% (UR 23-61%)) notifications in 2025. Impacts of both the retreatment and up-front interventions on MDR-TB incidence (and mortality) were slightly greater among these simulations than among the full set of simulations, reflecting the greater transmission resulting from each untreated case and the faster rate of TB and thus drug-resistance diagnosis in these simulations compared to those with longer average active-TB duration (S1 Table).

The relatively small additional gains achieved by MDR-TB diagnosis and treatment in new, rather than only previously-treated, patients in the initial analysis reflect relatively small ensuing reductions in infectious time combined with low overall MDR-TB treatment success rates. The probability of cure for a case of MDR-TB diagnosed under the primary intervention, after accounting for initial loss to follow-up, treatment discontinuation, treatment failure, and relapse, is only 49% (UR 32-69%). Additionally, among those treatment-naïve MDR-TB patients who are treated and cured, eliminating the six-month delay of ineffective first-line treatment reduces average infectiousness-weighted duration of disease by only 23% (UR 13-32%). Allowing for improved second-line cure rates when MDR-TB patients were TB-treatment-naïve (i.e. reducing their median probability of bacteriologic treatment failure from 25% to 12.5%) improved the median probability of non-spontaneous cure for a patient diagnosed with MDR-TB from 49% to 57%, given unchanged rates of death, loss to follow up during treatment, relapse, and initial loss to follow-up. Considering also our assumption of needing five years of linear scale-up to be able to offer DST and second-line treatment to all new patients, this modeled increase in bacteriologic response to MDR-TB treatment when patients were TB-treatment-naïve resulted in only a slight improvement in the impact of the up-front DST and treatment intervention (S1 Table).
