# Supplementary material for: MDR-TB treatment as prevention: The projected population-level impact of expanded treatment for multidrug-resistant tuberculosis
Source: PLoS One. 2017 Mar 8;12(3):e0172748. doi: 10.1371/journal.pone.0172748 (PMC5342197; doi:10.1371/journal.pone.0172748)
Supplement: S2 Text — Annotated differential equations specifying transmission model. (PDF) [file pone.0172748.s002.pdf]

Model Equations

Disease states

(Denotes the fraction of the population in a given state or set of states)

S = susceptible (never-infected, or cured/recovered)
L = latently infected
E = early active TB (not yet seeking care)
A = active TB (not yet diagnosed or on treatment)
B_{1e} = on effective first-line treatment (leading to culture conversion)
B_{1i} = on ineffective first-line treatment (remain infectious)
B_{2e1} = on initial six months of effective second-line treatment
B_{2e2} = on continuation phase of effective second-line treatment
B_{2i} = on ineffective second-line treatment
C = known active MDR-TB not being treated
W = asymptomatic but will relapse
X = any of the above states
I = any infectious state

Subscripts denoting more specific states

Treatment status: $T = N$ (new) or P (previously diagnosed with and/or treated for TB)
Drug resistance: $D = S$ (drug-susceptible) or R (drug-resistant)
Note: When T or D is used as index in a sum, $\bar{T}$ and $\bar{D}$ denote the opposite treatment status or drug resistance state from the one being indexed.

Parameters:

t = time in years from year 2000

i_X = infectiousness (and also mortality) of state X, relative to active untreated TB

β_0 = transmission coefficient in year 2000 (chosen to give year 2000 incidence when run to equilibrium)

d = rate of linear decline in transmission coefficient β_S, derived by fitting linear model to overall TB incidence in 2000 and 2013

f_R = transmission fitness of MDR strain, relative to $f_S = 1$ for DS strain, in year 2000

e = relative rate of decline in β_R, as fraction of d

μ = baseline mortality rate of adults age > 15 years without TB

μ_{tb} = excess mortality rate of active untreated TB

ν = rate of spontaneous cure from active TB

τ_{t1} = duration of first-line therapy

τ_{t21} = duration of second-line therapy, initial phase

τ_{t22} = duration of second-line therapy, continuation phase

$\sigma_{1N\ P}$ = first-line treatment success rate – fraction of adherent (new, previously-treated) DS-TB patients who convert to culture-negative on first-line therapy

σ_2 = second-line treatment success rate – fraction of adherent MDR-TB patients who convert to culture-negative on second-line therapy

$\delta_{1N,P}$ = fraction of (new, previously-treated) patients lost to follow up during first-line therapy

δ_2 = fraction of patients lost to follow up during second-line therapy

$\alpha_{1N,P}$ = fraction of (new, previously-treated) DS-TB patients who acquire drug resistance during fist-line therapy

$\omega_{1N,P}$ = fraction of (new, previously-treated) DS-TB patients who will relapse after completing first-line therapy

ω_2 = fraction of MDR-TB patients who will relapse after completing second-line therapy

ω_δ = fraction of DS-TB patients who will relapse if lost to follow up during effective first-line therapy

τ_ω = mean time to relapse, among patients who will relapse

$\gamma_{N,P}$= fraction of (new, previously-treated) patients who are immediately retreated after failing first-line therapy, if not diagnosed with MDR-TB

ρ = probability of rapid progression to (early) active disease after infection

λ = reduction in probability of rapid progression from superinfection, if already latently infected

r = rate of reactivation (latent to early active)

a = rate of progression from early-active to fully-active TB

$x_{N,P}$ = rate of diagnosis and first-line treatment initiation or drug susceptibility testing (DST) in (new, previously-treated) patients

$s_{pN,pP}$ = fraction of (new, previously-treated) patients receiving DST prior to treatment initiation

$s_{fN,fP}$ = fraction of (new, previously-treated) patients receiving DST at the end of a failing regimen

b = fraction, of patients diagnosed with MDR-TB, who receive second-line treatment

Time-dependent quantities:

Transmission coefficient:

$\beta_S(t) = \beta_0 - d \cdot t$
Observed declines in TB incidence are modeled as a linear decline over time in the transmission coeffienct. (Sensitivity analyses consider an alternative model in which the decline in incidence is due to decreased reactivation from latency.)

$\beta_R(t) = (\beta_0 - ed \cdot t) f_R$
Transmissibility of the drug-resistant strain may be less than that of the drug susceptible strain, and transmission of this strain also decreases over time in the primary analysis.

Note: For new individuals entering the population at age 15, the fraction with latent TB is dependent on the average transmission coefficient over the preceding 15 years, $\beta_D(t - \frac{15}{2})$.

Force of infection:

$FOI_D(t) = \Sigma_{X \in I_D}(X(t) i_X \beta_D(t))$

At any given point in time, all individuals in infectious states contribute additively to the force of infection. When their infectious state is less infectious than fully active, untreated TB, their contribution to the force of infection is reduced by factor i_X.

Differential equations:

Susceptible states

$$\frac{dS_N}{dt} = \left[\sum_X (\mu + i_X \mu_{tb}) X(t)\right] e^{-\sum_D \sum_{X \in I_D} i_X X(t) \beta_D (t - \frac{15}{2})} + \nu(\sum_D (E_{ND}(t) + A_{ND}(t))) - (\mu + \sum_D FOI_D(t)) S_N(t) \tag{1}$$

Susceptibles (uninfected) with no history of prior TB treatment are generated by replacement of natural and TB-related deaths with new 15 year olds; the fraction of these 15 year olds who are susceptible rather than latently infected is based on the force of infection over the previous 15 years.
Spontaneous resolution of early-active and active TB prior to starting treatment also results in return to the never-treated susceptible compartment. Losses from this compartment occur due to natural death and due to infection.

$$\frac{dS_P}{dt} = \nu \left(\sum_D (E_{PD}(t) + A_{PD}(t)) + \sum_{T,D} (B_{1eTD}(t) + B_{1iTD}(t)) + \sum_T (B_{2e1T}(t) + B_{2e2T}(t)) + C_R(t) \right) + \sum_T \left[B_{1e(TS)}(t)(1 - \omega_{1T} - \delta_{1T}\omega_\delta)/\tau_{t1} \right] + B_{2e2}(t)(1 - \omega_2)/\tau_{t22} - (\mu + \sum_D FOI_D(t)) S_P(t) \tag{2}$$

Spontaneous resolution of TB at rate ν in previously-treated patients with recurrent early-active, active, or chronic TB or in individuals on effective or ineffective first- or second-line treatment places them in the susceptible, previously-treated (i.e. cured) compartment.
So does treatment-related cure, at the end of a treatment course of duration τ, except for the fractions ω who will relapse. Losses from this susceptible compartment can occur due to death or infection.

Latently infected states

$$\begin{aligned}\frac{dL_{ND}}{dt} &= \left[\sum_X (\mu + i_x\mu_{tb})X(t)\right] \frac{\sum_{X\in I_D} i_X X(t)\beta_D(t-\frac{15}{2})}{\sum_D \sum_{X\in I_D} i_X X(t)\beta_D(t-\frac{15}{2})} \left(1 - e^{-\sum_D \sum_{X\in I_D} i_X X(t)\beta_D(t-\frac{15}{2})}\right) \\ &+ (1-\rho)FOI_D(t)S_N(t) \\ &+ (1-(1-\lambda)\rho)\left[\frac{f_D}{\sum_D f_D} FOI_D(t)L_{N\bar{D}}(t) - \frac{f_{\bar{D}}}{\sum_D f_D} FOI_{\bar{D}}(t)L_{ND}(t)\right] \\ &- (1-\lambda)\rho FOI_D(t)\sum_D L_{ND}(t) - (r+\mu)L_{ND}(t)\end{aligned} \tag{3}$$

$$\begin{aligned}\frac{dL_{PD}}{dt} &= (1-\rho)FOI_D(t)S_P(t) \\ &+ (1-(1-\lambda)\rho)\left[\frac{f_D}{\sum_D f_D} FOI_D(t)L_{P\bar{D}}(t) - \frac{f_{\bar{D}}}{\sum_D f_D} FOI_{\bar{D}}(t)L_{PD}(t)\right] \\ &- (1-\lambda)\rho FOI_D(t)\sum_D L_{PD}(t) - (r+\mu)L_{PD}(t)\end{aligned} \tag{4}$$

As new 15 year olds replace natural and TB-related deaths, the fraction who enter the population with latent infection is determined by cumulative exposure to each strain over the past 15 years; these have no history of treated (are added to the L_N states only).
New latent infections for both new and previously-treated states also arise from exposure of susceptibles (who progress rapidly with probability ρ or otherwise become latently infected),
or of latently-infected individuals (whose probability ρ of rapid progression is reduced by factor $1-\lambda$).
Losses from the latently-infection compartments occur due to super-infection with rapid progression (with probability $(1-\lambda)*\rho$), from reactivation (rate r), and from natural mortality (rate μ).

Early active disease

$$\frac{dE_{TD}}{dt} = \rho S_T(t)FOI_D(t) + rL_{TD} + (1-\lambda)\rho FOI_D(t)\sum_D L_{TD}(t) - (x_T + \nu + \mu + i_E\mu_{tb})E_{TD}(t) \tag{5}$$

Early active disease develops after initial infection with rapid progression, reactivation from latency, superinfection with rapid progression, or relapse following successful treatment. Losses from this compartment occur from further progression to fully-active TB, from spontaneous resolution, and from natural or TB-related mortality.

Active disease

$$\frac{dA_{NS}}{dt} = aE_{NS}(t) - (x_N + \nu + \mu + \mu_{tb})A_{NS}(t) \tag{6}$$

For all strains and all treatment histories, active disease develops via progression from early active disease (E), and losses from these active disease compartments occur due to treatment initiation, spontaneous resolution, and natural or TB-related death.

$$\frac{dA_{PS}}{dt} = aE_{PS}(t) + (1/\tau_\omega)W_S(t) + \frac{1}{\tau_{t1}}\sum_T((1-\alpha_T)B_{1iTS}(t)) - (x_P + \nu + \mu + \mu_{tb})A_{PS}(t) \tag{7}$$

Additional previously-treated, drug-susceptible active disease cases arise from relapses and from treatment failures without acquired drug resistance.

$$\frac{dA_{NR}}{dt} = aE_{NR}(t) - (x_N + \nu + \mu + \mu_{tb})A_{NR}(t) \tag{8}$$

$$\begin{aligned}\frac{dA_{PR}}{dt} &= aE_{PR}(t) + \sum_T\left(\frac{(1-s_{fT})(1-\gamma_T)}{\tau_{t1}}(B_{1iTR}(t) + \alpha_T B_{1iTS}(t))\right) \\ &- (x_P + \nu + \mu + \mu_{tb})A_{PR}(t)\end{aligned} \tag{9}$$

Additional previously-treated, drug-resistant active cases arise from treatment failures (second-line treatment failures, or first-line treatment failures who acquire resistance), who are not immediately retreated.

Chronic active untreated MDR-TB

$$\frac{dC_R}{dt} = (1-b)\sum_T \left(s_{pT}x_T A_{TR}(t) + \frac{s_{fT}}{\tau_{t1}}(B_{1iTR}(t) + \alpha_T B_{1iTS}(t)) \right) \\ + \frac{1}{\tau_{t21}}(B_{2i}(t) + \delta_2 B_{2e1}(t)) + 1/\tau_\omega W_R(t) \\ - (\nu + \mu + \mu_{tb})C_R(t) \tag{10}$$

We assume that individuals who are diagnosed with MDR-TB but do not initiate treatment, who fail MDR-TB treatment or are lost to follow up during MDR-TB treatment, or who relapse after MDR-TB treatment, are not retreated for MDR-TB but instead move to a chronic active disease compartment where they remain until spontaneous cure or death.

Treatment states

$$\frac{dB_{1eNS}}{dt} = x_N \sigma_{1N} A_{NS}(t) - (\nu + \mu + i_{B_{1e}}\mu_{tb} + 1/\tau_{t1})B_{1eNS}(t) \tag{11}$$

As active drug-susceptible cases (A) are diagnosed and start treatment at rate x, a fraction σ experience initial bacteriologic response to treatment and enter the effective first-line treatment (B_{1e}) states.
Individuals may leave this and all other treatment states due to spontaneous resolution, baseline or TB-related mortality, or completion of the treatment course.

$$\frac{dB_{1ePS}}{dt} = x_P \sigma_{1P} A_{PS}(t) + (\sigma_{1P}\tau_{t1})\sum_T [\gamma_T(1-\alpha_T)B_{1iTS}(t)] - (\nu + \mu + i_{B_{1e}}\mu_{tb} + 1/\tau_{t1})B_{1ePS}(t) \tag{12}$$

In addition to new treatment initiations in previously-treated individuals with drug-susceptible active disease, patients who are failing treatment for drug-susceptible TB and have not acquired resistance may be retreated and respond to retreatment.

$$\frac{dB_{1iNS}}{dt} = x_N(1-\sigma_{1N})A_{NS}(t) - (\nu + \mu + i_{B_{1i}}\mu_{tb} + 1/\tau_{t1})B_{1iNS}(t) \tag{13}$$

For the other $1-\sigma$ fraction of patients, treatment is ineffective despite being correctly chosen based on their drug-susceptible disease status.

$$\frac{dB_{1iPS}}{dt} = x_P(1-\sigma_{1P})A_{PS}(t) + \frac{1-\sigma_{1P}}{\tau_{t1}}\sum_T [\gamma_T(1-\alpha_T)B_{1iTS}(t)] - (\nu + \mu + i_{B_{1i}}\mu_{tb} + 1/\tau_{t1})B_{1iPS}(t) \tag{14}$$

Previously-treated patients without MDR have a higher average probability σ_P than treatment-naive patients of failing treatment.

$$\frac{dB_{1iNR}}{dt} = x_N(1-s_{pN})A_{NR}(t) - (\nu + \mu + i_{B_{1i}}\mu_{tb} + 1/\tau_{t1})B_{1iNR}(t) \tag{15}$$

If new TB patients are diagnosed with TB but their drug resistance is not diagnosed, they initiate ineffective first-line therapy.

$$\frac{dB_{1iPR}}{dt} = x_P(1-s_{pP})A_{PR}(t) + \sum_T \frac{(1-s_{fT})\gamma_T}{\tau_{t1}}[B_{1iNR}(t) + \alpha_{1N}B_{1iNS}(t)] - (\nu + \mu + i_{B_{1i}}\mu_{tb} + 1/\tau_{t1})B_{1iPR}(t) \tag{16}$$

Previously-treated patients with drug-resistant TB may similarly start ineffective first line therapy when they are diagnosed with TB, when they complete one ineffective course of first-line therapy and still receive no DST, or after they acquire resistance during treatent for drug-susceptible disease and receive no DST before retreatment.

$$\frac{dB_{2e1}}{dt} = b\sigma_2 \sum_T \left[s_{pT}x_T A_{TR}(t) + \frac{s_{fT}}{\tau_{t1}}(B_{1iTR}(t) + \alpha_T B_{1iTS}(t)) \right] - (\nu + \mu + i_{B_{2e1}}\mu_{tb} + \frac{1}{\tau_{t21}})B_{2e1}(t) \tag{17}$$

Of MDR TB patients who are appropriately diagnosed with drug resistance when they are initiating therapy or after they fail due to inappropriate treatment or newly acquired drug resistance, a fraction b will initiate MDR therapy and a fraction σ of those will respond to treatment.

$$\frac{dB_{2e2}}{dt} = (1-\delta_2)\frac{1}{\tau_{t21}}B_{2e1}(t) - (\nu + \mu + i_{B_{2e2}}\mu_{tb} + \frac{1}{\tau_{t22}})B_{2e2}(t) \tag{18}$$

Those who respond to MDR treatment may be lost to follow up at time τ_{t21}, but those not lost to follow up complete therapy after time τ_{t21}.

$$\frac{dB_{2i}}{dt} = b(1-\sigma_2)\sum_T \left[s_{pT}x_T A_{TR}(t) + \frac{s_{fT}}{\tau_{t1}}(B_{1iTR}(t) + \alpha_T B_{1iTS}(t)) \right] - (\nu + \mu + i_{B_{2i}}\mu_{tb} + \frac{1}{\tau_{t21}})B_{2i}(t) \tag{19}$$

Those MDR patients who do not respond to attempts at MDR treatment for a time time τ_{t21} subsequently move to chronic active disease.

States pending relapse

$$\frac{dW_S}{dt} = \sum_T \frac{\omega_T + \delta_T \omega_\delta}{\tau_{t1}} B_{1eTS}(t) - (\mu + \frac{1}{\tau_\omega})W_S(t) \quad (20)$$

A specified fraction of individuals who successfully complete treatment, and a larger fraction of those lost to follow up prior to completing treatment, will relapse to active disease after a time τ_ω unless they die first.

$$\frac{dW_R}{dt} = \frac{\omega_2}{\tau_{t22}} B_{2e2}(t) + \frac{1}{\tau_{t1}} \sum_T \alpha_T(\omega_T + \omega_\delta \delta_T) B_{1eTS}(t) - (\mu + \frac{1}{\tau_\omega})W_R(t) \quad (21)$$

Relpasing MDR cases can occur after MDR treatment or after acquisition of resistance during first-line treatment of drug-susceptible disease.
