# Supplementary material for: MDR-TB treatment as prevention: The projected population-level impact of expanded treatment for multidrug-resistant tuberculosis
Source: PLoS One. 2017 Mar 8;12(3):e0172748. doi: 10.1371/journal.pone.0172748 (PMC5342197; doi:10.1371/journal.pone.0172748)
Supplement: S1 Table — (DOCX) [file pone.0172748.s003.docx]

**S1 Table: Sensitivity Analysis - Comparison with alternative model assumptions.**

| **Intervention** | **Response variable (measure of year-2025 MDR-TB incidence)**^1^ | **Model**^2^ | | | | |
| --- | --- | --- | --- | --- | --- | --- |
|  |  | ***Original model*** | ***Declining reactivation rate*** | ***Reduced infectiousness on ineffective treatment*** | ***Reduced MDR failure if treatment-naïve*** | ***Shorter-active-TB-duration subset of simulations*** |
| **Current care** | Absolute incidence (/100K person-yr) | 7.4 (3.0-19.4) | 6.9 (3.6-16.4) | 6.7 (4.1-11.4) | 7.4 (3.0-19.4) | 7.1 (3.0-18.1) |
| **Primary intervention**^3^ | Absolute incidence (/100K person-yr) | 5.2 (2.5-13.6) | 4.4 (2.6-10.5) | 4.9 (3.2-7.6) | 5.2 (2.5-13.6) | 4.8 (2.4-12.1) |
|  | Percent reduction (vs standard care) | 26 (4-52) % | 33 (11-61) % | 27 (9-41) % | 26 (4-52) % | 29 (8-56) % |
| **Alternative intervention**^4^ | Absolute incidence (/100K person-yr) | 5.0 (2.4-13.1) | 4.2 (2.5-10.0) | 5.0 (3.2-7.7) | 4.9 (2.4-12.8) | 4.6 (2.4-11.7) |
|  | Percent reduction (vs standard care) | 29 (6-55) % | 36 (14-64) % | 26 (7-40) % | 30 (7-56) % | 32 (9-59) % |

^1^All results are reported as median (95% uncertainty range).

^2^The alternative models are described in the supplementary methods (S1 Text)

^3^Primary modeled intervention is drug susceptibility testing in all retreatment patients by 2017 and initiation of MDR treatment for 85% of those diagnosed

^4^Alternative intervention is drug susceptibility testing of all retreatment patients by 2017 *and* all new patients by 2020, again with treatment initiation for 85% of those diagnosed with MDR
