# Supplementary figures and images for: MDR-TB treatment as prevention: The projected population-level impact of expanded treatment for multidrug-resistant tuberculosis
Source: PLoS One. 2017 Mar 8;12(3):e0172748. doi: 10.1371/journal.pone.0172748 (PMC5342197; doi:10.1371/journal.pone.0172748)

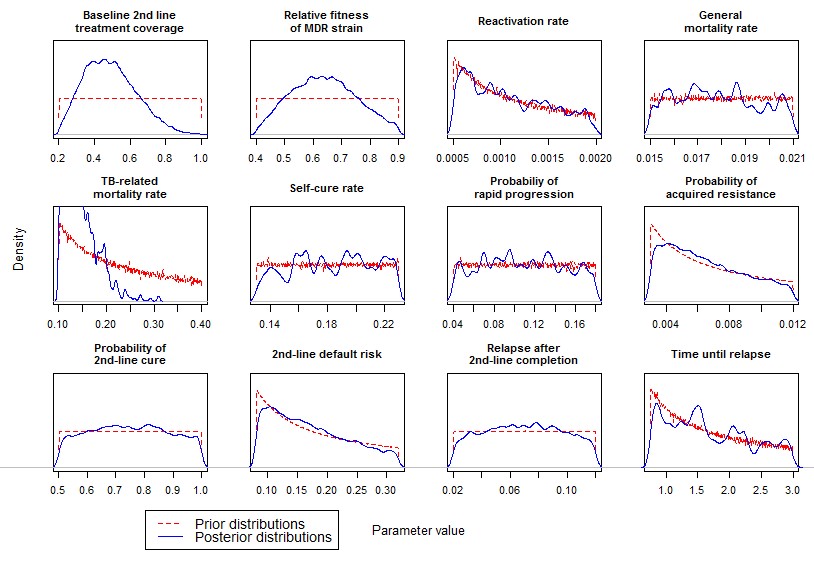

Supplement: S1 Fig — Prior distributions (among all parameters sets generated for consideration by Latin hypercube sampling) and posterior distributions (among epidemic trajectories that were accepted as consistent with present-data notification data) of key model parameters. Most parameters’ sampled ranges were represented evenly among the accepted simulations (blue curves, most of which are similar to the red), but a few parameters (notably the extent of second-line treatment coverage and the relative fitness of the MDR strain) exerted greater influence on the fit of simulations to notification data. (JPG) [file pone.0172748.s004.jpg]
